# The Safety of Public Water Supplies

**Published:** 1922-02

**Authors:** 


### THE SAFETY OF PUBLIC WATER SUPPLIES.

**With Special Reference to the "Wallace-Tiernan" Chlorinator.**

THE potency of chlorine as an agent of water sterilisation was conclusively demonstrated in the European War, one of the most remarkable features of which was the fact that throughout the operations on the Western Front there was no epidemic of any water-borne diseases. Bearing in mind that in a large number of instances the ordinary sources of supply were either rendered useless or totally destroyed, and that to make up the deficiency drinking water for the armies had to be taken haphazard from streams, canals and rivers, the entire success of the measures employed for the sterilisation of the supplies is obvious.

Of all the methods which were tried at the outbreak of war, treatment by free chlorine gained the most satisfactory results. In the beginning, the application of the element in the form of bleaching powder was favoured on account of the difficulties in dealing with the free gas, but the disadvantages of using "bleach" were found to be considerable owing to the difficulties in handling and the variations in strength and its deterioration by keeping. In the meantime, the "Wallace-Tiernan" apparatus for adding the gas directly had been brought to a high pitch of perfection, and was eventually employed by all the Allied Armies as the standard chlorinator, with the results which are well known. The experience thus gained has a far-reaching effect on present-day methods of purifying public supplies.

It is now twenty years since Sir Alexander Houston proved the beneficial effects arising from storage, and although nothing that has happened in the intervening period has caused him to depart from the opinion he then formed, the practical difficulties of erecting large storage reservoirs in many cases militates against the adoption of this method of purification. The lime treatment, which was also introduced by Sir Alexander Houston with the object of lessening the time required for storage, has been tried in one or two places, but requires careful control and is not altogether satisfactory as a mode of sterilisation for general purposes.

Chlorine fulfils every requirement of the water engineer and chemist, providing its administration is rigidly controlled in a reliable apparatus. In chlorination two main conditions have to be fulfilled. Economy is the first, and it has been proved that chlorine gas is more economical to use than bleaching powder. Certainty of action is the second, and on this point the evidence is overwhelming. Not only the experiences of the war, but the established successs of chlorine treatment in over one hundred cities and towns in the United States, has put the efficacy of chlorine beyond all possibility of doubt.

The "Wallace-Tiernan" chlorinator, to which reference has been made, is an appliance which has proved adequate to meet the widely varying requirements of handling chlorine in the liquid or gaseous state for purification of water or sewage. Chlorine gas compressed to the liquid state in iron cylinders, holding 100 lb., is controlled and measured by the apparatus and introduced into the water or sewage in the proper proportion to effect sterilisation. There are two general types of apparatus, one by which the chlorine is introduced directly into the water or sewage, and the other by which the gas is first dissolved in a small quantity of water and the resulting chlorine solution piped to the point of application. The first type is called the "Dry Feed," and the second the "Wet Feed." Each type is furnished for either manual or automatic control. The chlorine meter operates on the hydraulic principle, and has no mechanical parts. Each apparatus is empirically calibrated, and the flow of the chlorine is made visible by an ingenious arrangement.

Several English towns are using this apparatus, which has also been adopted by the municipality of New York for the purification of the entire water supply of New York City, amounting to 340 million gallons daily. This is the largest chlorinating equipment in the world, but it should be pointed out that the cost of the chlorinator is moderate, and that it can be economically applied for the effective sterilisation of the smallest as well as of the largest quantities of water.

